# MSR2N: Multi-Stage Rotational Region Based Network for Arbitrary-Oriented Ship Detection in SAR Images

**DOI:** 10.3390/s20082340

**Published:** 2020-04-20

**Authors:** Zhenru Pan, Rong Yang, Zhimin Zhang

**Affiliations:** 1 Space Microwave Remote Sensing System, Aerospace Information Research Institute, Chinese Academy of Sciences, Beijing 100190, China; yangrong16@mails.ucas.ac.cn (R.Y.); zmzhang@mail.ie.ac.cn (Z.Z.); 2 School of Electronic, Electrical and Communication Engineering, University of Chinese Academy of Sciences, Beijing 100039, China

**Keywords:** synthetic aperture radar (SAR) ship detection, multi-stage rotational region based network (MSR2N), rotated anchor generation, multi-stage rotational detection network (MSRDN)

## Abstract

In synthetic aperture radar (SAR) images, ships are often arbitrary-oriented and densely arranged in complex backgrounds, posing enormous challenges for ship detection. However, most existing methods detect ships with horizontal bounding boxes, which leads to the redundancy of detected regions. Furthermore, the high Intersection-over-Union (IoU) between two horizontal bounding boxes of densely arranged ships can cause missing detection. In this paper, a multi-stage rotational region based network (MSR2N) is proposed to solve the above problems. In MSR2N, the rotated bounding boxes, which can reduce background noise and prevent missing detection caused by high IoUs, are utilized to represent ship regions. MSR2N consists of three modules: feature pyramid network (FPN), rotational region proposal network (RRPN), and multi-stage rotational detection network (MSRDN). First of all, the FPN is applied to combine high-resolution features with semantically strong features. Second, in RRPN, a rotation-angle-dependent strategy is employed to generate multi-angle anchors which can represent arbitrary-oriented ship regions more felicitously than horizontal anchors. Finally, the MSRDN with three sub-networks is proposed to regress proposals of ship regions stage by stage. Meanwhile, the incrementally increasing IoU thresholds are selected for resampling positive and negative proposals in sequential stages of MSRDN, which eliminates close false positive proposals successively. With the above characteristics, MSR2N is more suitable and robust for ship detection in SAR images. The experimental results on SAR ship detection dataset (SSDD) show that the MSR2N has achieved state-of-the-art performance.

## 1. Introduction

Ship detection is one of the most significant missions of marine surveillance. With the characteristics of working all-weather, all-time [1], and imaging relatively wide areas at constant resolution [2], synthetic aperture radars (SAR) such as Terra-X, COSMOS-SkyMed, RADARSAT-2, Sentinel-1, and GF-3 are widely applied in ship detection [3,4,5,6,7].

Traditional ship detection methods are mainly based on the following three aspects: (1) statistics characteristics [8,9,10,11]; (2) wavelet transform [12,13]; and (3) polarization information [14,15]. Among these methods, constant false alarm rate (CFAR) and variants thereof [8,9,10] are most widely utilized. CFAR detectors adaptively calculate the detection thresholds by estimating the statistics of background clutter and maintain a constant probability of false alarm. However, the determination of detection thresholds depends on the distribution of sea clutter, which is not robust enough for the detection of multi-scale ships in multi-scenes. On the other hand, CFAR based methods require land masking and post-processing to reduce false alarms, which is insufficiently automated.

Recently, with the rapid development of deep convolutional networks [16,17,18,19,20], great progress has been made in deep convolutional neural networks (CNN)-based object detection [21,22,23,24,25,26,27,28,29,30,31,32,33]. Generally, the current CNN-based detectors can be divided into one-stage [25,26,29,31] and two-stage detectors [22,23,24,28,32,33]. One-stage detectors include you only look once (YOLO) [25] and its derivative versions [34,35], single shot detector (SSD) [26] and RetinaNet [29], et al. YOLO reframes object detection as a regression problem. The input images are divided into S×S grid cells and then YOLO predicts bounding boxes and class probabilities for each grid cell straightly. SSD generates a set of default boxes over different sizes per feature map location to match the shape of objects better. RetinaNet proposed the focal loss to overcome the extreme foreground-background class imbalance. On the other hand, faster region-based CNN (Faster R-CNN) [23] and region-based fully convolutional networks (R-FCN) [28] are representative two-stage detectors. Faster R-CNN generates anchors of different scales and aspect ratios through the region proposal network (RPN). In addition, then the feature map and proposals rescaled from anchors are fed into the Fast R-CNN sub-network to predict the location and class probabilities of bounding boxes. Different from the per-region sub-network of Faster R-CNN, R-FCN is a fully convolutional network with the shared computation on the entire image. It proposed position-sensitive score maps to address the problem between translation-invariance in image classification and object detection [28]. Feature pyramid network (FPN) [24] combines low-level and high-level features for more comprehensive feature expression, which has outstanding performance on multi-scale object detection. In summary, one-stage detectors show superiority in detection speed benefits from the single network of detection pipeline. However, for accuracy, the two-stage detectors are better than that of one-stage, especially for small dense object detection.

For SAR ship detection, Deep CNNs have been widely applied in recent years. As a typical one-stage detection method, YOLOv2 was utilized to detect ships in SAR imagery [36]. Wang et al. [37] utilized RetinaNet for automatic ship detection of multi-resolution GF-3 imagery. Zhang et al. [38] proposed a lightweight feature optimizing network with lightweight feature extraction and attention mechanism for better feature representation. On the other hand, many two-stage detectors were proposed for higher detection accuracy. Ref. [39] proposed an improved Faster R-CNN for SAR ship detection. A multilayer fusion light-head detector [40] was proposed to improve the detection speed. Jiao et al. [41] proposed a densely connected neural network, which utilizes a modified FPN, for multi-scale and multi-scene ship detection. Ref. [42,43,44,45] added attention mechanisms into CNNs as attention mechanisms adaptively recalibrate feature responses to increase representation power [19,46].

**Figure 1:**
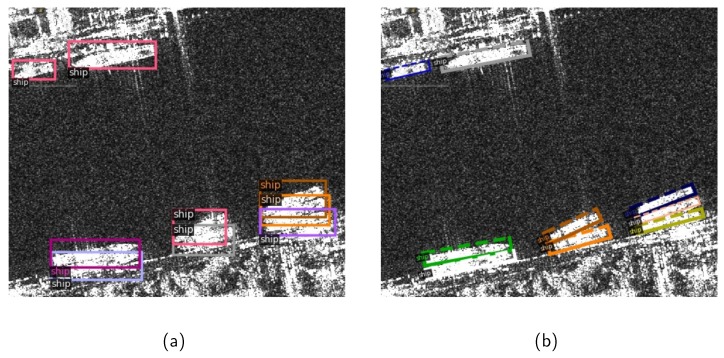
Densely arranged ships in an inshore complex background. (**a**) marking ship regions with horizontal bounding boxes; (**b**) marking ship regions with rotated bounding boxes.

**Figure 2:**
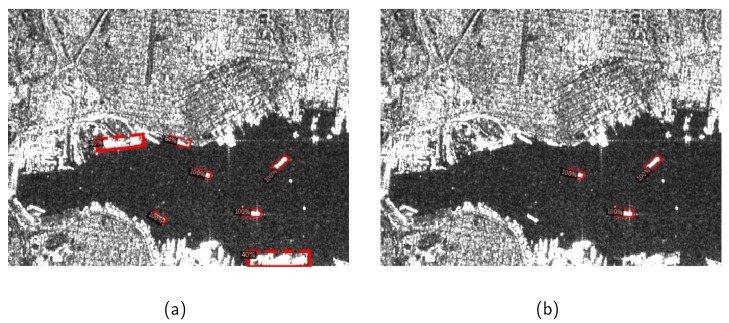
Detection results under different IoU thresholds; (**a**) detection results under a IoU threshold of 0.5; (**b**) detection results under a IoU threshold of 0.7.

Though many CNN-based methods have been proposed for SAR ship detection, they still encounter bottlenecks on the following issues: (1) Quite different from natural images, in SAR images, strip-like ships are often presented under bird’s eye perspectives with various rotation angles and densely arranged in an inshore complex background, as shown in Figure 1a. A ship with an inclined angle leads to a relatively large redundancy region, which would introduce background noise. Moreover, two horizontal bounding boxes of densely arranged ships have a high Intersection-over-Union (IoU) leading to missing detection after the non-maximum suppression (NMS) operation [47,48]. Under these circumstances, the limited capacity of detection with horizontal bounding boxes would be exposed. (2) In R-CNN based object detection methods, an IoU threshold is utilized to distinguish positive and negative samples in the Fast R-CNN sub-network. A relatively low IoU threshold will result in a high recall but a low precision due to the generation of noisy bounding boxes. On the contrary, a relatively high IoU threshold leads to inadequate positive samples. In this case, the overfitting model will cause missing detection. Figure 2 shows the detection results under IoU thresholds of 0.5 and 0.7, respectively. Some noisy background regions are detected as ship regions in Figure 2a, and the missing detection appears in Figure 2b.

Due to the inherent drawback of horizontal bounding boxes, rotated bounding boxes were gradually developed in optical remote sensing [47,48,49,50]. Ref. [47] proposed a rotation dense feature pyramid network (R-DFPN) in which the dense FPN and multiscale region of interest (ROI) align are used to detect ships in different scenes. In [48], a multi-category rotation detector was proposed for small, cluttered and rotated objects. Li et al. [49] proposed a rotatable region-based residual network (R3-Net) for multi-oriented vehicle detection. The ROI Transformer [50] that is lightweight was proposed to decrease the computational complexity.

To address the problems in SAR ship detection, a multi-stage rotational region based network (MSR2N) is proposed for arbitrary-oriented ship detection. As shown in Figure 1b, rotated bounding boxes can locate ships more accurately with less redundant noise background, and would not overlap with each other even in a dense arrangement. Therefore, the rotated bounding box representation is utilized in this paper. In the feature extraction module, we apply the FPN to fuse the high-resolution features and semantically strong features from the backbone network, which enhances feature representation. To generate rotated anchors and proposals, a rotational RPN (RRPN) is utilized. In addition, a multi-stage rotational detection network (MSRDN) is applied in MSR2N. The MSRDN, which contains an initial rotational detection network (IRDN) and two refined rotational detection networks (RRDN), is trained stage by stage. Three increasing IoU thresholds are selected to sample the positive and negative proposals in three stages, respectively. The increasing IoU thresholds guarantee sufficient positive samples to avoid overfitting, and reduce close false positive proposals in the meantime. Compared to other methods, the proposed MSR2N achieves state-of-the-art performance on SAR ship detection, especially for densely arranged ships in inshore complex backgrounds. The main contributions of proposed MSR2N are enumerated as follows:

1. Alluding to the characteristics of SAR images, the MSR2N framework is proposed in this paper, which is more beneficial for arbitrary-oriented ship detection than horizontal bounding box based methods.
2. In RRPN, a rotation-angle-dependent strategy is utilized to generate anchors with multiple scales, ratio aspects, and rotation angles, which can represent arbitrary-oriented ships more adequately.
3. The MSRDN is proposed, where three increasing IoU thresholds are chosen to resample and refine proposals successively. With the proposals refined more accurately, the number of refined proposals also increases.
4. The multi-stage loss function is employed to accumulate losses of RRPN and three stages of MSRDN to train the entire network.
5. Compared to other methods, the proposed MSR2N has achieved state-of-the-art performance on SAR ship detection dataset (SSDD).

This paper is organized as follows. Section 2 describes the proposed MSR2N in detail. In Section 3, the ablation and comparative experiments are carried out on SSDD, which verifies the effectiveness of proposed MSR2N. Section 4 draws the conclusions for this paper.

## 2. Proposed Approach

**Figure 3:**
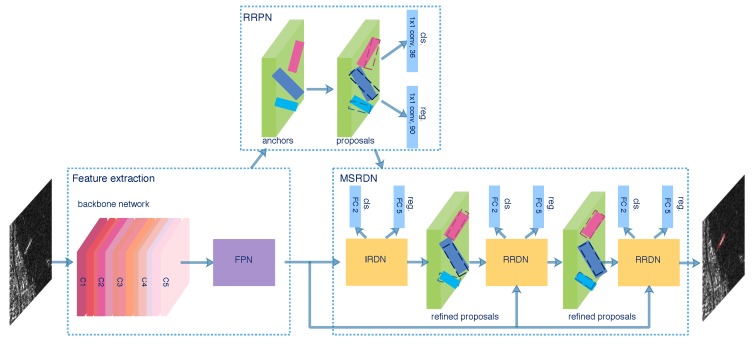
Overall framework of MSR2N.

The overall framework of the proposed MSR2N is illustrated in Figure 3. The whole framework can be divided into three modules: FPN, RRPN, and MSRDN. The FPN fuses feature maps from different layers of the backbone to generate the feature pyramid. In the RRPN module, a rotated anchor generation strategy is utilized to produce anchors with various rotation angles. The RRPN head outputs the softmax probabilities of being a ship and the regression offsets that encode the coordinates of coarse proposals. Millions of proposals are generated, but most of them are redundant noisy proposals and the amount of computation is huge. Hence, we select $N_{pre}$ proposals with the highest probabilities per feature pyramid level and perform the skew NMS operation [51] on the selected proposals to obtain $N_{post}$ final proposals which are fed into the next MSRDN. The MSRDN containing the IRDN and two RRDNs is trained stage by stage with three increasing IoU thresholds. During training, the MSR2N is optimized under the supervision of softmax probabilities and rotated boxes regression offsets from RRPN and each stage of MSRDN. For the inference time, the final refined proposals from the last stage of MSRDN are selected by a probability threshold and sampled by the skew NMS operation. Finally, the predicted bounding boxes are obtained.

### 2.1. Feature Pyramid Network

**Figure 4:**
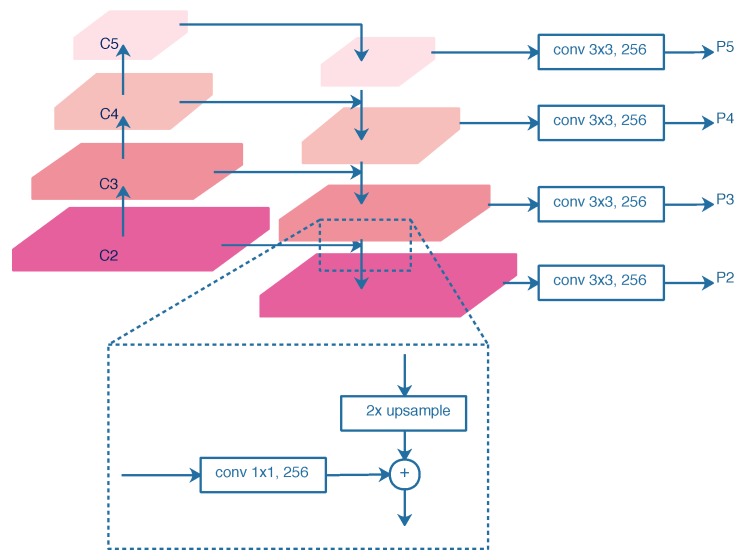
Architecture of FPN.

In this paper, we select ResNet50 [17] as the backbone network, as shown in Figure 3. To extract sufficient ship features in SAR images, especially for small ships, the FPN [24] is utilized to combine the high-resolution, semantically weak features with low-resolution, and semantically strong features. As illustrated in Figure 4, the FPN contains a bottom-up pathway, a top-down pathway, lateral connections, and output convolutions. The outputs of ResNet50s last residual blocks, denoted as {C2, C3, C4, C5}, are chosen as the bottom-up hierarchy. The strides of {C2, C3, C4, C5} are {4, 8, 16, 32} pixels with respect to the input image. The top-down pathway upsamples the semantically stronger features by a factor of 2 and fuses them with the spatially finer features from the bottom-up pathway by element-wise addition. The lateral connections are 1 × 1 convolution layers with 256-channels, which reduces channel dimensions. In addition, 3 × 3 convolution layers with 256-channels are utilized as the output layers to reduce the aliasing effect of upsampling. The final feature maps of FPN denoted as {P2, P3, P4, P5}, are fed into the following stages.

### 2.2. Parameterization of Rotated Bounding Box

**Figure 5:**
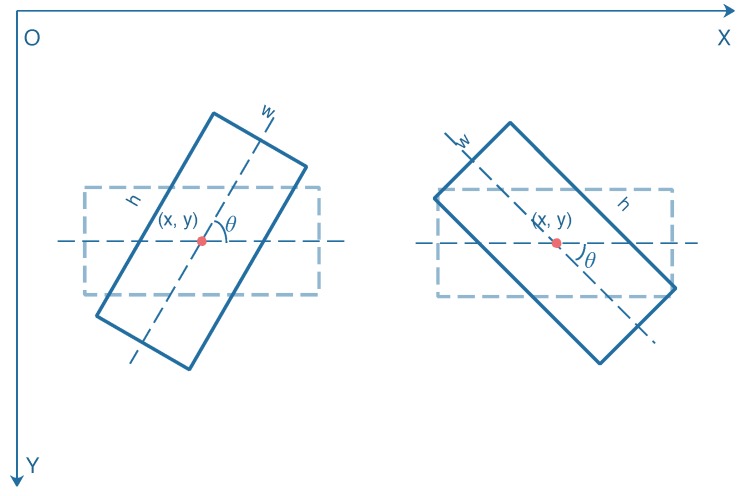
Geometric representation of two rotated bounding boxes. The rotation angle θ of the bounding box on the left is $60^{\circ }$, and the rotation angle θ of the bounding box on the right is $-45^{\circ }$.

In the conventional SAR ship detection networks, the ship region is a horizontal rectangle, which is represented by four parameters ($x_{min},y_{min},x_{max},y_{max}$). ($x_{min},y_{min}$) and ($x_{max},y_{max}$) denote the coordinates of the top left and bottom right corners of a bounding box, respectively. For a rotated bounding box, five parameters (x,y,w,h,θ) are conducted. The coordinate (x,y) represents the center of the rotated bounding box. To avert the disorder of coordinate expression, we set *w* and *h* as the longer side and shorter side of the rotated bounding box. θ denotes the angle from the positive direction of the *x*-axis counterclockwise to the longer side of the rotated bounding box. As half angular space is enough for the description of the rotation angle, the range of θ is set to $\left[-90^{\circ },90^{\circ }\right)$. Figure 5 shows the geometric representation of two rotated bounding boxes with different rotation angles.

### 2.3. Rotated Anchors and Proposals

**Figure 6:**
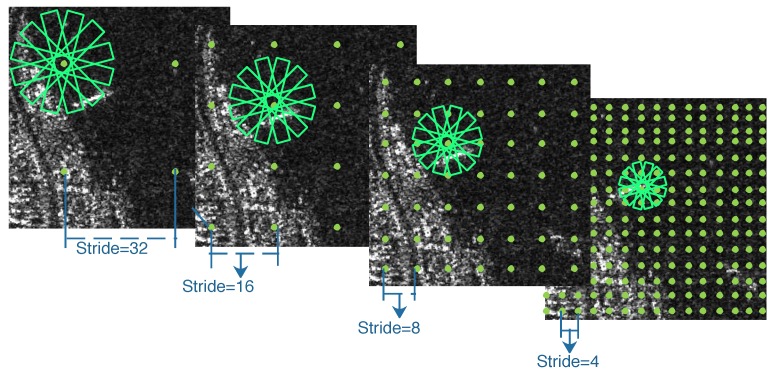
Anchor generation in an SAR image.

**Figure 7:**
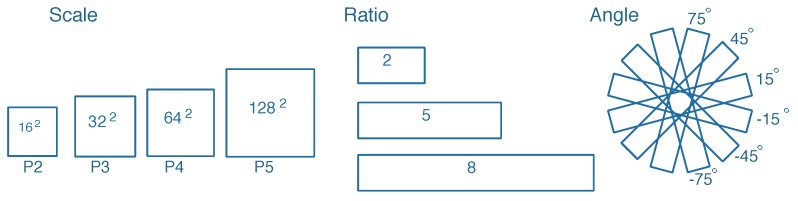
Cell anchor generation.

In [24], multi-scale anchors with multiple aspect ratios are generated on the SAR images with respect to the feature pyramid. However, the generation of horizontal anchors is not sufficient enough for arbitrary-oriented bounding boxes. On the other hand, the characteristic of ships with large aspect ratios should be taken into consideration. Corresponding to the representation of rotated bounding boxes, we utilize a rotation-angle-dependent strategy to generate rotated anchors. As illustrated in Figure 6, a set of cell anchors are generated at the points of a SAR image with the strides of FPN, respectively. As shown in Figure 7, three aspects are taken into consideration in the cell anchor generation. First, referring to [24], the scales of anchors are set to {$16^{2},32^{2},64^{2},128^{2}$} pixels on the feature pyramid levels of {P2, P3, P4, P5}, respectively. Second, the large aspect ratios {2, 5, 8} replace the usual aspect ratios {0.5, 1, 2} in [23,24] with a view to the characteristic of strip-like ships. Finally, we set multiple rotation angles for anchors of various scales and aspect ratios, which can represent proposals more effectively. Six rotation angles {−75°,−45°,−15°,15°,45°,75°} are set in consideration of the trade-off between half angular coverage and computational efficiency. Hence, each point per level of feature pyramid generates 18 (1 × 3 × 6) anchors. As shown in Figure 3, the classification layer outputs 36 (2 × 18) softmax probabilities of being a ship and background, and the regression layer outputs 90 (5 × 18) regression offsets that encode the coordinates (x,y,w,h,θ) for 18 proposals per position.

### 2.4. Multi-Stage Rotational Detection Network

#### 2.4.1. Initial Rotational Detection Network

As shown in Figure 3, the feature pyramid {P2, P3, P4, P5} and the coarse proposals produced from RRPN are both fed into the next stage called IRDN. Different from the conventional object detection network, IRDN aims to detect rotated proposals in this paper. $N_{RROI}$ proposals are randomly sampled as an RROI mini-batch in which an IoU threshold is chosen to distinguish the positive and negative samples. Here, we utilize the skew IoU computation [51] to compute IoU between rotated bounding boxes. If the IoU between a proposal and any ground-truth box is higher than the threshold, the proposal is assigned to a positive sample and vice versa. The ratio of positive and negative proposals is 1:3. In the RROI mini-batch, if the number of positive proposals is less than 25% of $N_{RROI}$, the negative proposals should be padded.

**Figure 8:**
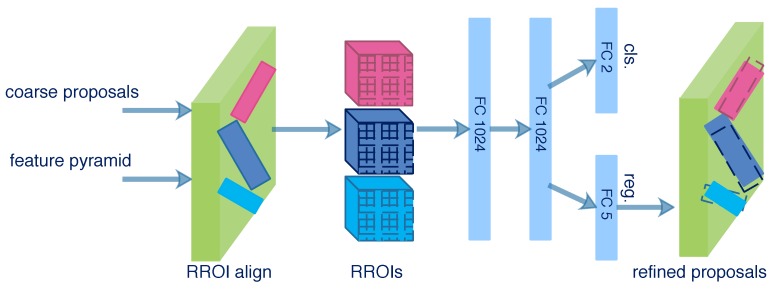
Structure of IRDN.

As illustrated in Figure 8, the rotational ROI (RROI) align layer is applied for arbitrary-oriented proposals. It converts the feature map of RROIs into fixed spatial feature maps. An RROI is divided into 7 × 7 sub-regions by the RROI align layer, and the max pooling is operated in each sub-region. Then, the feature map of an RROI with a fixed spatial extent of 7 × 7 can be obtained. The feature maps of RROIs are input to two successive fully connected (FC) layers with a dimension of 1024. Two 1 × 1 convolutional layers for classification and regression are attached to the FC layers. The classification layer outputs softmax probabilities of being a ship and background, and the regression layer outputs the regression offsets that encode the coordinates (x,y,w,h,θ) of refined proposals.

#### 2.4.2. Multi-Stage Detector

As mentioned in Section 1, a single IoU threshold in IRDN can’t separate positive and negative samples properly, which can cause false and missing detection. Inspired by [52], a multi-stage rotational detection strategy is proposed. As illustrated in Figure 3, the MSRDN contains an IRDN and two RRDNs which share the same structure with IRDN. The MSRDN is a multi-stage regression framework, which successively resamples proposals by increasing IoU thresholds. In the first stage of MSRDN, the distribution of coarse proposals is set by a low IoU threshold, which guarantees enough positive samples. The feature pyramid and sampled coarse proposals are fed into the IRDN to produce the refined proposals. In the second stage, the refined proposals from the first stage are resampled by a medium IoU threshold. These sampled refined proposals and the feature pyramid are fed into the RRDN which outputs new refined proposals. In the third stage, the repetitive procedures occur with a high IoU threshold. The process of MSRDN can be expressed as:

(1) $$\begin{array}{c} MSRDN\left(f,d^{1}\right)=IRDN\left(f,d^{1}\right)\circ RRDN_{1}\left(f,d^{2}\right)\circ RRDN_{2}\left(f,d^{3}\right) \end{array}$$

where *f* denotes the feature maps from FPN, $d^{1}$ is the initial sampled distribution of proposals in IRDN, $d^{2}$ and $d^{3}$ are respectively the resampled distribution of proposals in two RRDNs, and ∘ represents the cascade operation. The sub-networks are optimized by the resampled distributions $\left\{d^{1},d^{2},d^{3}\right\}$, respectively. With a sequence of increasing IoU thresholds, the number of close false positive proposals are sequentially decreased, and the rotated bounding boxes regression is more accurate.

### 2.5. Multi-Stage Loss Computation

During the training of RRPN, a sampling strategy for rotated anchors is used. A mini-batch of $N_{RRPN}$ anchors is selected for the loss computation, where the ratio of positive and negative samples is 1:1. In the mini-batch, if positive samples are not enough, the negative samples should be padded. The positive samples are defined by following rules: (i) the IoU between the anchor and any ground-truth is larger than 0.7; and (ii) an angular difference between the anchor and the ground-truth is smaller than $15^{\circ }$. The negative samples should satisfy the following rules: (i) the IoU between the anchor and any ground-truth is lower than 0.3; or (ii) the IoU between the anchor and a ground-truth is larger than 0.7, while the angular difference is larger than $15^{\circ }$. We minimize an objective function of RRPN with the multi-task loss [22] defined as:

(2) $$\begin{array}{c} L_{RRPN}\left(p_{i},p_{i}^{*},t_{i},t_{I}^{*}\right)=\frac{1}{N_{cls}}\sum_{i}L_{cls}\left(p_{i},p_{i}^{*}\right)+\lambda \frac{1}{N_{reg}}\sum_{i}p_{i}^{*}L_{reg}\left(t_{i},t_{i}^{*}\right). \end{array}$$

Here, *i* denotes the index of samples in a mini-batch, $p_{i}^{*}$ is the label of sample *i*, where $p_{i}^{*}$ equals 1 if the sample is a positive one and $p_{i}^{*}$ equals 0 if the sample is a negative one. $p_{i}$ is the predicted softmax probablility of sample *i* being a ship. $t_{i}^{*}$ represents the offsets between sample *i* and the assigned ground-truth, and $t_{i}$ represents the predicted bounding box regression offsets. In Equation (2), only if sample *i* is a positive one with $p_{i}^{*}=1$ is the regression loss $L_{reg}$ calculated. The classification loss $L_{cls}$ is log loss which is defined as:

(3) $$\begin{array}{c} L_{cls}\left(p_{i},p_{i}^{*}\right)=-logp_{i}p_{i}^{*}. \end{array}$$

The regression loss is defined by the robust loss function smooth L1 as:

(4) $$\begin{array}{c} L_{reg}\left(t_{i},t_{i}^{*}\right)={smooth}_{L1}\left(t_{i}-t_{i}^{*}\right), \end{array}$$

(5) $$\begin{array}{c} {smooth}_{L1}\left(x\right)=\left\{\begin{array}{cc} 0.5x^{2},\; & |x|<1\\ |x|-0.5,\; & otherwise \end{array}\right.. \end{array}$$

The classification loss $L_{cls}$ and regression loss $L_{reg}$ are respectively normalized by $N_{cls}$ and $N_{reg}$, where $N_{cls}$ and $N_{reg}$ are both set to the mini-batch size. The regression loss $L_{reg}$ is weighted by a balancing parameter λ, which is set to 1 in the experiments.

For rotated bounding box regression, the five parameterized coordinate regression offsets are defined as:

(6) $$\begin{array}{c} t_{x}=\left(x-x_{a}\right)/w_{a},\;t_{y}=\left(y-y_{a}\right)/h_{a},\\ t_{w}=log\left(w/w_{a}\right),\;t_{h}=log\left(h/h_{a}\right),\\ t_{\theta }=\theta -\theta_{a}+k\pi ,\\ t_{x}^{*}=\left(x^{*}-x_{a}\right)/w_{a},\;t_{y}=\left(y^{*}-y_{a}\right)/h_{a},\\ t_{w}^{*}=log\left(w^{*}/w_{a}\right),\;t_{h}^{*}=log\left(h^{*}/h_{a}\right),\\ t_{\theta }^{*}=\theta^{*}-\theta_{a}+k\pi , \end{array}$$

where x,y,w,h and θ denote the center coordinates, width, height, and rotation angle of a rotated bounding box. $x,x_{a},x^{*}$ are respectively for the predicted box, anchor box, and ground-truth box, likewise for y,w,h,θ. The parameter k∈Z keeps θ in the range of [−180°,180°).

As described in Section 2.4, during the training of MSRDN, the proposals are resampled into a mini-batch and refined by a sequence of rotational detection sub-networks. Meanwhile, each sub-network outputs the softmax probabilities of a proposal being a ship and rotated bounding box regression offsets for loss computation. The multi-task multi-stage loss function of MSRDN is defined as:

(7) $$\begin{array}{c} L_{MSRDN}=L_{IRDN}+L_{{RRDN}_{1}}+L_{{RRDN}_{2}} \end{array}$$

where $L_{IRDN}$, $L_{{RRDN}_{1}}$, and $L_{{RRDN}_{2}}$ represent the losses of IRDN, first RRDN and second RRDN, respectively. The losses of three stages have the same definition as the multi-task loss function of RRPN.

## 3. Experiments and Results

### 3.1. Dataset

We evaluate the proposed framework on the public SAR ship detection dataset—SSDD [39]. The SSDD contains SAR ship images of various scenes from three sensors. The details of SSDD are shown in Table 1. There are 1160 SAR images including 2456 ships in total, with an average of 2.12 ships per image. Ships in SSDD are annotated with coordinates of the four vertices of rotated bounding boxes. The SSDD is divided into a training set and a test set by a ratio of 4:1.

### 3.2. Setup and Implementation Details

The experiments are implemented in the deep learning framework Pytorch [53] on four NVIDIA TITIAN Xp GPUs with 12 GB memory. Our network is initialized by the pre-trained ResNet50 model for ImageNet classification. The whole network is trained for 12 k iterations in total, and a batch of 48 images is fed into the network in an iteration. The initial learning rate is 0.01 for the first 8k iterations; then, learning rates of 0.001 and 0.0001 are set for the next 2.5 k iterations and the remaining 1.5 k iterations, respectively. The stochastic gradient descent (SGD) [54] optimizer with a weight decay of 0.0001 and a momentum of 0.9 is selected for the model.

Referring to faster R-CNN, we resize images such that the shorter side is 350 pixels under the premise that ensures the longer side less than 800 pixels. For data augmentation, we flip the images horizontally with a probability of 0.5.

As mentioned in Section 2, $N_{pre}$ denotes the number of pre-selected proposals per feature pyramid level, and $N_{post}$ represents the number of post-selected proposals. Here, we set $N_{pre}$ to 2000 and 1000 for training and inference, respectively. In addition, $N_{post}$ is set to 1000 for both training and inference. $N_{RRPN}$ and $N_{RROI}$, which are the sizes of RRPN and RROI mini-batches, are set to 256 and 512, respectively. In inference time, an NMS threshold is used to select final predicted bounding boxes, and a predicted bounding box can be retained if its score is higher than the score threshold. The NMS threshold and score threshold are respectively set to 0.3 and 0.1 in all experiments.

### 3.3. Evaluation Metrics

To quantitatively evaluate the performance of proposed MSR2N, the precision, recall, mean Average Precision (mAP), and F-measure (F1) are utilized as evaluation metrics.

Precision is the rate that correctly detected ships in all detected results, and recall means the rate that correctly detected ships in all ground-truths. The definitions of precision and recall are as follows:

(8) $$\begin{array}{c} Precision=\frac{TP}{TP+FP} \end{array}$$

(9) $$\begin{array}{c} Recall=\frac{TP}{TP+FN} \end{array}$$

where TP, FP, and FN denote the number of correctly detected ships, false alarms, and undetected ships, respectively. In addition, a bounding box is admitted as a correctly detected ship in the case that the IoU between the bounding box and a ground-truth is higher than the threshold of 0.5. The precision–recall (PR) curve shows the precision–recall pairs at different confidence score thresholds.

The mAP is a comprehensive metric that calculates the average value of precision under the recall in a range of [0, 1]. The definition of mAP is as follows:

(10) $$\begin{array}{c} mAP=\int_{0}^{1}P\left(R\right)dR \end{array}$$

where *R* denotes a recall value and *P* represents the precision corresponding to a recall.

F1 evaluates the comprehensive performance of a detector by taking the precision and recall into consideration. F1 is defined as:

(11) $$\begin{array}{c} F1=\frac{2*Precision*Recall}{Precision+Recall}. \end{array}$$

### 3.4. Ablation Study

In this part, some ablation experiments are carried out to investigate the effectiveness of the main modules of the proposed MSR2N.

#### 3.4.1. Effect of MSRDN

To illustrate the detection performance of MSRDN, the experimental results are listed in Table 2. We can find that the single-stage detectors have poor detection performance. The detector with a low IoU threshold of 0.5 achieves a high recall but a low precision due to the introduction of noisy regions. With the increase of the IoU threshold, the precision increases, but the recall decreases. When the IoU threshold reaches 0.7, the recall decrease to 84.50% because of the overfitting caused by inadequate positive proposals. When using the two-stage detectors with an IRDN and an RRDN, they can achieve better detection performance than the single-stage ones. The detection performance of three-stage detector with the increasing IoU thresholds of {0.5, 0.6, 0.7} is significantly improved, which achieves the recall of 92.05%, precision of 86.52%, mAP of 90.11%, and F1 of 89.20%. These experiments demonstrate the effectiveness of multiple stages of MSRDN. Comparing to the three-stage detectors with uniform thresholds, the detector with the increasing IoU thresholds still has superior performance, which proves the effectiveness of increasing IoU thresholds of MSRDN. With the multi-stage regression, the number of close false positive proposals is decreased and the location of proposals is more accurate in the meantime. Therefore, the MSRDN with the increasing IoU thresholds of {0.5, 0.6, 0.7} is chosen in this paper.

#### 3.4.2. Effect of Multi-Stage Loss Computation

The experimental results of multi-stage loss computation are presented in Table 3. It can be observed that the detection performance with three-stage loss computation is preferable, and the experiment only using loss from the last stage obtains the inferior detection performance. With the multi-stage loss used, the precision increases significantly, which means that the false alarms are effectively suppressed. We can conclude that the multi-stage loss computation has a strong constraint on the training of the whole network.

#### 3.4.3. Effect of Rotation Angles

Some experiments about rotation angles are carried out, and their results are shown in Table 4. It can be seen in Table 4 that the method which only generates horizontal and vertical anchors achieves inferior detection performance. As the interval of rotation angles decreases, the detection performance improves. The method with the rotation angles of {−75°,−45°,−15°,15°,45°,75°} obtains superior detection performance, which proves the effectiveness of anchors with different rotation angles.

#### 3.4.4. Effect of FPN

A couple of ablation experiments are performed to demonstrate the effect of FPN in the proposed MSR2N, wherein the experiment of MSR2N uses the feature pyramid {P2, P3, P4, P5} as the outputs of feature extraction module, and the experiment of MSR2N without FPN only uses C5 of ResNet50 to feed into the next stages. Table 5 shows the experimental results of the FPN. Comparing to MSR2N without FPN, our MSR2N reaches higher evaluation metrics. The significant improvement of detection performance benefits from the sufficient feature expression of FPN.

### 3.5. Comparison with Other Object Detection Methods

To verify the performance of our proposed MSR2N, we compare the MSR2N with the multi-stage horizontal region based network (MSHRN), rotational Faster R-CNN [23] (Faster RR-CNN), rotational FPN [24] (R-FPN), and rotational RetinaNet [29] (R-RetinaNet). MSHRN is the horizontal variant of our proposed MSR2N, where the RPN sub-network is used and the ROI pooling layer replaces the RROI align layer for predicting horizontal bounding boxes. Faster RR-CNN and R-FPN are two-stage detectors, which are respectively rotational variants of Faster R-CNN and FPN. The one-stage detector R-RetinaNet is the rotational variant of RetinaNet. In [29], the feature pyramid with levels {P3, P4, P5, P6, P7} is used as the output of the feature extraction module. For consistency, we use the feature pyramid {P2, P3, P4, P5} in the experiment of R-RetinaNet, which is the same as the experiments of R-FPN and MSR2N. Here, ResNet50 is chosen as the backbone network of all methods.

**Figure 9:**
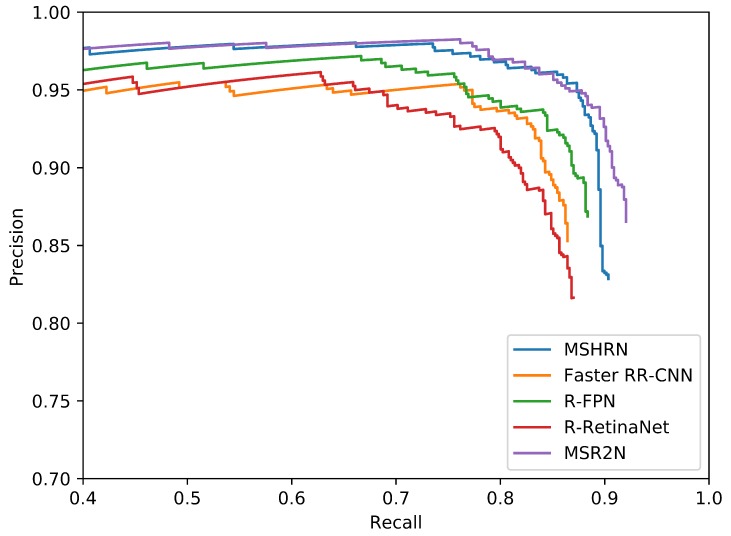
PR curves of different methods on SSDD.

Table 6 summarizes the experimental results of different methods on SSDD, and Figure 9 shows the PR curves of different methods. From Table 6, we can find that MSR2N achieves state-of-the-art performance: 92.05% for recall, 86.52% for precision, 90.11% for mAP, and 89.20% for F1. Comparing to the other four methods, MSR2N obtains 2.27%, 7.89%, 6.45%, and 9.54% gains in mAP, respectively. In Figure 9, the PR curve of MSR2N is higher than those of Faster RR-CNN, R-FPN, and R-RetinaNet. The PR curve of MSHRN only can reach a recall of 90.36%, but MSR2N can achieve a recall of 92.05%. This phenomenon indicates that horizontal region based detection leads to more undetected ships than rotational region based detection. To further verify the capability of handling hard cases, we construct a subset by selecting SAR images, which contain densely arranged ships in inshore complex scenes, from the original test set. Table 7 shows the experimental results of different methods on hard cases. From Table 7, it can be observed that all the values of the evaluation metrics are relatively low, which indicates that these hard cases are difficult to detect correctly. For example, the mAP of R-RetinaNet only reaches 42.91%. Nevertheless, our proposed MSR2N achieves superior performance. Comparing to the other four methods, MSR2N obtains 12.43%, 8.52%, 13.08%, and 28.30% gains in mAP, respectively.

**Figure 10:**
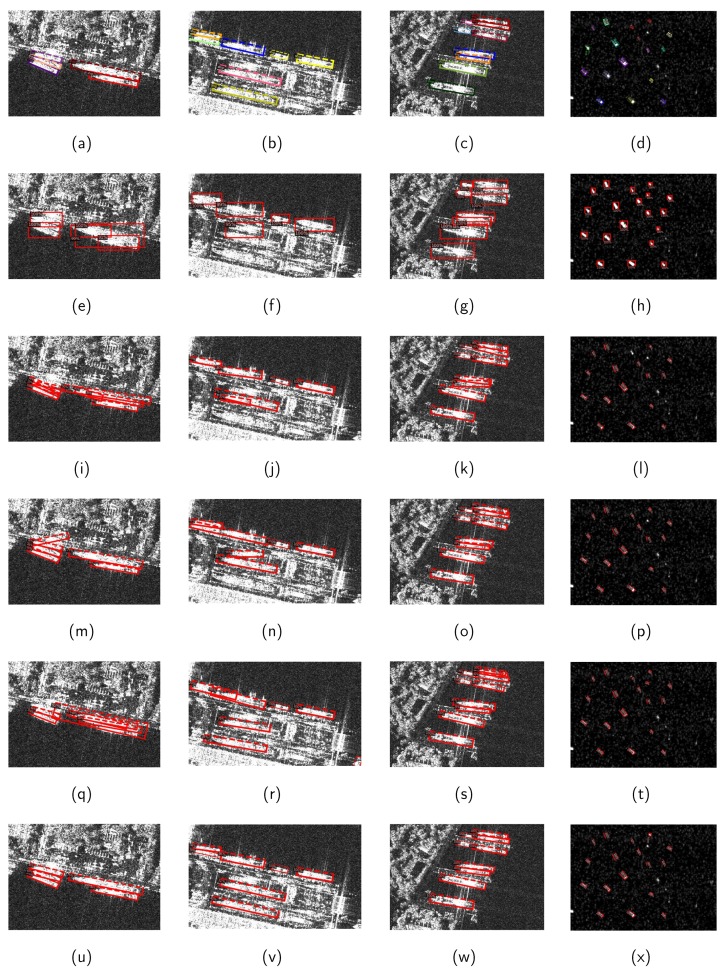
Detection results of different methods. (**a**–**d**) ground-truths; (**e**–**h**) detection results of MSHRN; (**i**–**l**) detection results of Faster RR-CNN; (**m**–**p**) detection results of R-FPN; (**q**–**t**) detection results of R-RetinaNet; (**u**–**x**) detection results of MSR2N.

Figure 10 illustrates the detection results of four images from the test set of SSDD using different methods. From the detection results of three inshore SAR images, it can be observed that there exist undetected ships in Figure 10e–g. This is on account of the high IoU between two overlapped horizontal bounding boxes in MSHRN. On the other hand, the background noise in the horizontal bounding boxes can interfere with the detection. In addition, the detection results of Faster RR-CNN, R-FPN, and R-RetinaNet still have problems with missing detection and false detection. On the contrary, MSR2N shows superior detection performance in Figure 10u–w. All ships are successfully detected, and the position of ships is located more accurately than other methods. For the methods of MSHRN, Faster RR-CNN, and R-RetinaNet, there exist several false alarms and undetected ships in the results of fourth SAR images. Generally speaking, all methods are competent to detect ships far from shore. Based on the above discussion, we can conclude that MSR2N has state-of-the-art performance on SAR ship detection, especially for densely arranged ships in complex backgrounds.

## 4. Conclusions

This paper proposes an arbitrary-oriented ship detection network called MSR2N which aims at detecting ships in various complex scenes. In our MSR2N, the rotating bounding boxes are utilized to represent ship regions, which can reduce redundant noisy regions and overlaps between bounding boxes. The SSDD is employed to verify the effectiveness of the proposed MSR2N. The ablation experiments illustrate that high precision and high recall can be achieved simultaneously due to the sequential resampling of proposals and training in MSRDN sub-networks. The experiments about loss computation show the significance of multi-stage loss computation. The experiments of rotation angles and FPN are also carried out, which proves the effectiveness of rotation angles and FPN. Compared to other methods, MSR2N obtains superior detection performance, especially when ships are densely arranged in inshore complex scenes.

## Figures and Tables

**Table 1:** Detailed description of SSDD.

**Sensors**	RadarSat-2, TerraSAR-X, Sentinel-1
**Polarization**	HH, VV, HV, VH
**Sence**	inshore, offshore
**Resolution**	1 m–15 m
**Number of images**	1160
**Number of ships**	2456

**Table 2:** Experimental results of MSRDN.

Stage	IoU Thresholds	Recall (%)	Precision (%)	mAP (%)	F1 (%)
**IRDN**	{0.5}	89.53	86.03	83.66	87.75
	{0.6}	88.37	86.86	84.38	87.61
	{0.7}	84.50	**92.78**	83.09	88.44
**IRDN+RRDN1**	{0.5, 0.6}	90.70	85.56	87.57	88.05
	{0.5, 0.7}	89.53	86.68	84.88	88.08
	{0.6, 0.7}	89.53	88.68	86.78	89.10
**IRDN+RRDN1+RRDN2**	{0.5, 0.5, 0.5}	90.89	66.71	86.90	76.95
	{0.6, 0.6, 0.6}	90.70	85.25	88.55	87.89
	{0.7, 0.7, 0.7}	89.53	87.67	87.89	88.59
	{0.5, 0.6, 0.7}	**92.05**	86.52	**90.11**	**89.20**

**Table 3:** Experimental results of multi-stage loss computation.

Loss Computation	Recall (%)	Precision (%)	mAP (%)	F1 (%)
**RRDN2**	83.91	14.26	78.97	24.37
**RRDN1+RRDN2**	88.57	45.84	84.43	60.41
**IRDN+RRDN1+RRDN2**	**92.05**	**86.52**	**90.11**	**89.20**

**Table 4:** Experimental results of rotation angles.

Rotation Angles	Recall (%)	Precision (%)	mAP (%)	F1 (%)
**{$-90^{\circ },0^{\circ }$}**	88.92	53.33	85.50	66.67
**{$-90^{\circ },-45^{\circ },0^{\circ },45^{\circ }$}**	89.53	86.03	88.90	87.75
**{$-75^{\circ },-45^{\circ },-15^{\circ },15^{\circ },45^{\circ },75^{\circ }$}**	**92.05**	**86.52**	**90.11**	**89.20**

**Table 5:** Experimental results of FPN.

FPN	Recall (%)	Precision (%)	mAP (%)	F1 (%)
**MSR2N without FPN**	87.60	85.28	85.12	86.42
**MSR2N**	**92.05**	**86.52**	**90.11**	**89.20**

**Table 6:** Experimental results of different methods on SSDD.

Method	Recall (%)	Precision (%)	mAP (%)	F1 (%)
**MSHRN**	90.36	82.84	87.84	86.44
**Faster RR-CNN**	86.43	85.28	82.22	85.85
**R-FPN**	88.37	**86.86**	84.38	87.61
**R-RetinaNet**	87.01	81.64	82.80	84.24
**MSR2N**	**92.05**	86.52	**90.11**	**89.20**

**Table 7:** Experimental results of different methods on hard cases.

Method	Recall (%)	Precision (%)	mAP (%)	F1 (%)
**MSHRN**	61.54	60.61	58.78	61.07
**Faster RR-CNN**	56.76	70.00	62.69	51.04
**R-FPN**	63.51	75.81	58.13	69.12
**R-RetinaNet**	54.05	55.56	42.91	54.79
**MSR2N**	**76.12**	**77.37**	**71.21**	**76.74**
